# Mechanically Reinforced Catechol-Containing Hydrogels with Improved Tissue Gluing Performance

**DOI:** 10.3390/biomimetics2040023

**Published:** 2017-11-13

**Authors:** Jun Feng, Xuan-Anh Ton, Shifang Zhao, Julieta I. Paez, Aránzazu del Campo

**Affiliations:** 1 INM – Leibniz Institute for New Materials, Campus D2 2, 66123 Saarbrücken, Germany; Jun.Feng@leibniz-inm.de (J.F.); shifang.zhao@leibniz-inm.de (S.Z.); julieta.paez@leibniz-inm.de (J.I.P.); 2 Max-Planck-Institut für Polymerforschung, Ackermannweg 10, 55128 Mainz, Germany; xuananh.ton@gmail.com; 3 Chemistry Department, Saarland University, 66123 Saarbrücken, Germany

**Keywords:** tissue glues, reinforced hydrogels, bioinspired adhesives, catechol-functionalized polymers, nanocomposite

## Abstract

In situ forming hydrogels with catechol groups as tissue reactive functionalities are interesting bioinspired materials for tissue adhesion. Poly(ethylene glycol) (PEG)–catechol tissue glues have been intensively investigated for this purpose. Different cross-linking mechanisms (oxidative or metal complexation) and cross-linking conditions (pH, oxidant concentration, etc.) have been studied in order to optimize the curing kinetics and final cross-linking degree of the system. However, reported systems still show limited mechanical stability, as expected from a PEG network, and this fact limits their potential application to load bearing tissues. Here, we describe mechanically reinforced PEG–catechol adhesives showing excellent and tunable cohesive properties and adhesive performance to tissue in the presence of blood. We used collagen/PEG mixtures, eventually filled with hydroxyapatite nanoparticles. The composite hydrogels show far better mechanical performance than the individual components. It is noteworthy that the adhesion strength measured on skin covered with blood was >40 kPa, largely surpassing (>6 fold) the performance of cyanoacrylate, fibrin, and PEG–catechol systems. Moreover, the mechanical and interfacial properties could be easily tuned by slight changes in the composition of the glue to adapt them to the particular properties of the tissue. The reported adhesive compositions can tune and improve cohesive and adhesive properties of PEG–catechol-based tissue glues for load-bearing surgery applications.

## 1. Introduction

Tissue adhesives are promising biomaterials for wound closure, fixation of implants, and medical devices. Compared with sutures, tissue glues are less traumatic, and easier and faster to use, especially on soft tissues such as lung, liver, or kidney. Commercially available tissue glues can be classified into two categories: natural-based tissue adhesives including fibrin (Tisseel^®^ and Artiss^®^, Baxter; Evarrest^®^, Ethicon), albumin (BioGlue^®^, CryoLife), gelatin (FloSeal^®^, Baxter) or chitosan (Celox^®^, MedTrade); and synthetic tissue adhesives based on cyanoacrylate (Dermabond^®^, Ethicon; Leukosan^®^, BSN Medical; Histoacryl^®^, B. Braun), poly(ethylene glycol) (PEG) (CoSeal^®^, Baxter), or isocyanate (TissuGlu^®^, B. Braun), among others [1]. Several properties are desirable for a good tissue adhesive: strong adhesion in the presence of body fluids, biocompatibility, biodegradability, fast polymerization, adaptability to the mechanical properties of the tissue, and low cost. Cyanoacrylates and fibrin glues are among the most extensively used tissue adhesives. Despite this, none of them meets all requirements listed above [2]. Cyanoacrylate glues show high adhesion strength, rapid curing, and low cost. However, their degradation products are cytotoxic, making them unsuitable for internal use. Fibrin glues are biocompatible and biodegradable, but they exhibit poor mechanical strength. Considerable effort has been expended to develop in situ cross-linking hydrogels compatible with physiological conditions as alternative tissue gluing systems [3,4,5,6,7,8,9,10,11]. The main focus has been reinforcing interfacial properties by exploiting chemistries to improve reactivity with tissue [3]. Relevant outcomes in terms of interfacial strength in the presence of blood have been reported. However, all systems come up short for load-bearing applications in the clinic because of poor mechanical strength under physiological conditions [12,13].

Over the last few years, reinforced hydrogels with improved mechanical performance have been developed. Reinforcement can be achieved by mixing with micelles or micro- or nanoparticles that strongly interact with the polymer chains [14,15], with fibrillar nanostructures [16,17,18], or with other polymers [19,20]. Examples of PEG-based nanocomposite hydrogels include PEG diacrylate hydrogel containing hydroxyapatite [21] or Laponite [22] nanoparticles, or dopamine-functionalized 4-arm PEG hydrogel containing Laponite [23] or Fe_3_O_4_ [15] nanoparticles. Alternatively, double or interpenetrating networks combining two types of polymers have demonstrated impressive mechanical performance [14,24]. Examples of double networks include alginate/collagen-I mixtures for skin wound healing [25] or PEG diacrylate–collagen interpenetrated networks as cell scaffolds [26] and for vascular tissue engineering [27].

Catecholamine-functionalized polymers have attracted considerable attention as tissue adhesives in the last few years [3,10]. The idea of the catechol moiety as adhesive unit is inspired by the high content of dihydroxyphenylalanine (DOPA) amino acid of the adhesive mussel foot proteins (mfps) used by the mussel to attach to rocks in the sea. In tissue applications, oxidized catecholamines can undergo self-reaction (polymerization) or can bind to nucleophiles (such as –NH_2_ or –SH in the protein components of the tissue) under physiological conditions. This leads to rapid and effective cross-linking and tissue-binding of the catechol-derived material. A broad range of catecholamine-functionalized hydrogels have been reported, such as alginates [28,29], hyaluronan [30,31,32], heparin [33], gelatin [11], polyvinylpyrrolidone [34], PEG [8,35,36,37,38,39,40], and chitosan [10]. Reported applications of these materials include wound closure [35], hemostasis [10], adhesion in blood vessels [28], cell therapy [31,32], cell culture [33], biological glue [11], islet transplantation in the liver [8], or fetal membrane repair [37]. The catechol-based tissue glues show good interfacial strength in in vivo applications. However, as hydrogel-based materials, they present modest mechanical performance in wet environments, and their use in load-bearing applications is severely limited.

Cohesive and adhesive properties need always to be considered in the design of any adhesive, and optimized to the particular chemistry, mechanical properties, and mechanical solicitation of the glued parts. In this work, we present different formulations of mechanically robust PEG-based hydrogels and explore their potential as alternative matrices for tissue gluing with considerably better mechanical strength than reported systems. We describe adhesive networks based on collagen and PEG–catechol hydrogel, and hydrogel composites containing hydroxyapatite particles to improve the mechanical stability of the hydrogel matrix. The adhesion performance to pork skin was evaluated under different conditions: dry, wet, and in the presence of blood, and compared with reported and commercial systems. A biocompatible hydrogel composition was identified, with cohesive properties far better than PEG–catechol, both on wet skin and in the presence of blood. This system attained adhesion strength of ca. 40 kPa on skin tissue covered by blood, 6-fold higher than cyanoacrylate and 12-fold higher than fibrin.

## 2. Materials and Methods

### 2.1. Reagents and Materials

Tissues from pork (fresh skin) were obtained from a local butcher. The cyanoacrylate-based tissue adhesive Leukosan^®^ was obtained from BSN Medical (Hamburg, Germany). The fibrin-based tissue adhesive ARTISS^®^ was purchased from Baxter (Unterschleißheim, Germany). Collagen (type I, from rat tail, here referred to as Coll) and hydroxyapatite (nanopowder, <200 nm, referred to as HAp) were purchased from Sigma-Aldrich (Taufkirchen, Germany). 4-arm PEG succinimidyl carboxymethyl ester (PEG-NHS, M_w_ of 5, 10, and 40 kDa) was purchased from Jenkem Technology (Plano, TX, USA). All other reactants were purchased from Sigma-Aldrich and used as received. Poly(ethylene glycol) *O*,*O*’,*O*’’,*O*’’’-tetra(acetic acid dopamine) amide (PEG-Dop, Mw of 5, 10, and 40 kDa) were prepared following reported procedures from our group [40,41]. Details on the synthetic procedure and characterization (proton nuclear magnetic resonance (^1^H NMR) spectra, Supplementary Figure S1) can be found in the Supplementary Materials.

### 2.2. Preparation of Adhesive Formulations

For the preparation of pure PEG-Dop hydrogel, PEG-Dop was dissolved in 0.01 M phosphate-buffered saline (PBS, pH 7.4) at 10%, 20%, 30%, and 40% (*w*/*v*). An equivolume of NaIO_4_ solutions (concentration range 30–240 mM) in deionized (DI) water was added to induce gelation. The Dop:NaIO_4_ molar ratio was adjusted according to the specific requirements of experiments. For the adhesive test, 20 μL of the mixture of polymer and oxidant were spread on the skin. This amount was sufficient to cover 113 mm^2^ area of the tissue probe without significant overflow.

PEG-Dop/HAp: Nanocomposite hydrogels of PEG-Dop/HAp were prepared by adding the HAp nanoparticles suspension (prepared previously) to the PEG-Dop solution at 1%, 2%, 5%, and 10% (*w*/*v*). The mixture was vortexed and sonicated for 5 min to ensure homogeneity. The concentration of PEG-Dop was kept constant at 30% (*w*/*v*). Mixtures with NaIO_4_ solution were prepared and used as described above.

PEG-Dop/Coll and PEG-Dop/HAp/Coll: for the preparation of these two precursors, 1 M NaOH solution, 10× PBS, and DI water should be precooled and the whole mixing process should be performed on an ice bath. A collagen solution in 0.1 M acetic acid was neutralized with 1 M NaOH and 10× PBS to collagen concentrations of 0.1%, 0.2%, and 0.4% (*w*/*v*). PEG-Dop was added to the collagen solution and vortexed for 10 min to obtain a precursor of PEG-Dop/Coll (30% (*w*/*v*) PEG-Dop). For the PEG-Dop/HAp/Coll mixture, the HAp particles were first suspended in DI water by vortexing and sonicating for 10 min. The HAp suspension and PEG-Dop solution were added simultaneously to a previously neutralized collagen solution, and the mixture was vortexed and sonicated for 5 min for homogeneity. The pH of the resulting mixture was 7.0–7.4 and the concentration of the precursor was 0.2% (*w*/*v*) for collagen, 5% (*w*/*v*) for HAp, 30% (*w*/*v*) for PEG-Dop, and 1× for PBS. All collagen-containing systems were subsequently incubated at 37 °C for 3 h to allow collagen fibrillogenesis. Finally, the PEG-Dop/Coll and PEG-Dop/HAp/Coll network were mixed with an equivolume solution of 120 mM NaIO_4_ in DI water and applied to the tissue samples for adhesion tests as described above.

Collagen (0.2% (*w*/*v*)): Collagen (Type I, from rat tail) was diluted in 0.1 M acetic acid and neutralized with 1 M NaOH and 10× PBS to a collagen concentration of 0.2% (*w*/*v*) in 1× PBS at the pH of 7.0–7.4 (Note: precooled NaOH solution, 10× PBS, and DI water should be used, and the mixing should be performed in an ice bath). The collagen solution was kept at 37 °C to allow fibrillogenesis. After 3 h, 20 μL of the collagen solution was sandwiched between two skin disks and cured for 30 min at 37 °C.

Commercial tissue adhesives: 20 μL of cyanoacrylate or fibrin adhesive was applied to uniformly coat the skin surface without significant overflow, and cured between two disks of skin.

### 2.3. Adhesion Measurements

Fresh porcine skin was obtained from a local butcher and stored at −20 °C for a few months. When needed for the adhesion measurements, a portion of the tissue was defrosted and kept at room temperature in aluminum paper to prevent from drying. The defrosted tissue was used within a day.

For the adhesion measurements, porcine skin was cut into disks of 13 or 12 mm diameter with a hollow punch and the fat was removed with a scalpel. For adhesion measurements on wet skin, the skin disks were immersed in PBS for 2 min before adding the tissue glue on the skin covered by a thin layer of PBS. For adhesion measurements in the presence of blood, the skin sample was immersed in blood before the test. Blood was collected under informed consent from a healthy human donor, following the regulations and protocol approved by the ethic commission of the Ärztekammer des Saarlandes (EK-0001) and used within 10 min, or stored with heparin anticoagulants and kept at 4 °C for no longer than two days. No significant differences were found when the tests were performed with fresh blood or with heparin anticoagulants-treated blood.

Adhesion measurements were performed with a Zwick Roell 1446 Universal Testing Machine (Zwick Roell, Ulm, Germany) equipped with a 200 N load cell (Supplementary Figure S2). The protocol of adhesion test was adapted from the American Society for Testing and Materials (ASTM) standard F2258-05, to better suit our measurements. The main differences between our procedure and the ASTM standard were the shape of the skin samples (circular instead of squared, as we found circles were easier to cut precisely and reproducibly with a hollow punch), the pulling speed (1 mm/min instead of 2 mm/min, as the lower speed proved to be more reproducible in our hands), and the method for keeping the samples moist during the curing (no wet gauze was used, but was replaced by the specific conditions detailed below, in order to mimic application in clinics).

For measurements performed in dry conditions, cut skin disks of diameter 12 or 13 mm (contact area 113.04 or 132.7 mm², respectively) were immobilized with superglue on a “T” holder (Supplementary Figure S2). The adhesive mixture was deposited between the skin disks, a load of 0.2 N was applied and the adhesive was left for 30 min in the machine to allow complete gelation. The glue was always covering the whole area of the skin sample. For adhesion measurements on wet samples cured underwater, the samples were prepared as described before, but (i) the skin samples were immersed in PBS for 2 h and the flowing PBS was removed from the skin disks by tissue paper before applying the glue; and (ii) the skin–glue–skin sandwich was immersed in PBS for 30 min under a weight of 20 g for gelation. Then, the cured sample was glued to the machine for measurement. For adhesion measurements in presence of blood, blood was used instead of PBS and the curing was performed at 37 °C under a weight of 20 g, immersed in blood (approx. 5 mL was employed). The skin disks were pulled off at a rate of 1 mm/min.

**Figure 1:**
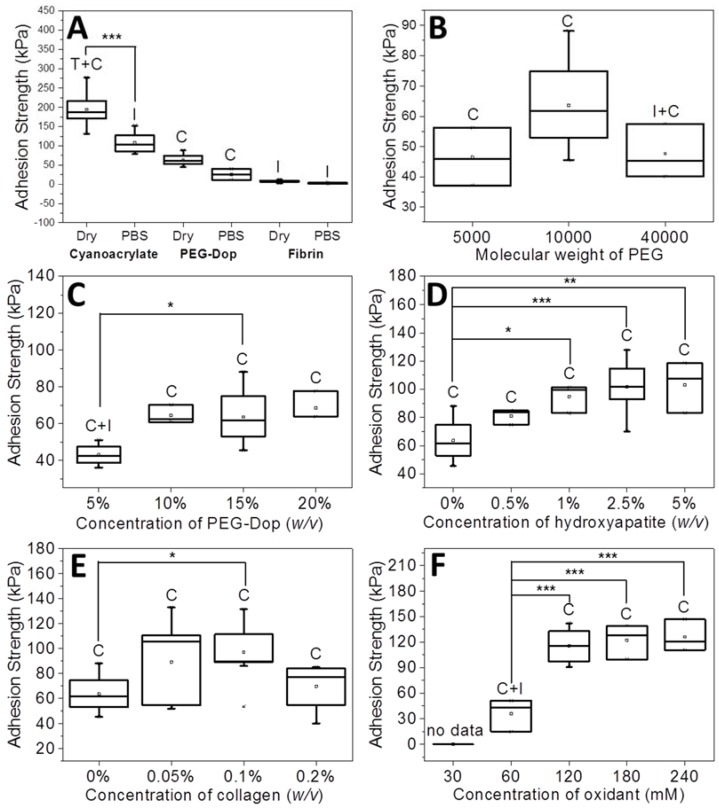
Adhesion strength of 10 kPa poly(ethylene glycol) *O*,*O*′,*O*′′,*O*′′′-tetra(acetic acid dopamine) amide (PEG-Dop)/NaIO_4_-based glues as function of the concentration of individual components in the gluing mixture. (**A**) Dry and wet test conditions (as compared with commercial tissue glues); (**B**) Molecular weight of poly(ethylene glycol) (PEG) (15% (*w*/*v*) PEG-Dop); (**C**) PEG-Dop concentration (120 mM NaIO_4_); (**D**) Hydroxyapatite (Hap) nanoparticle concentration (15% (*w*/*v*) PEG-Dop, 120 mM NaIO_4_); (**E**) Collagen (Coll) concentration (15% (*w*/*v*) PEG-Dop, 120 mM NaIO_4_); (**F**) Oxidant concentration (15% (*w*/*v*) PEG-Dop). Failure type: I, interfacial; C, cohesive; C + I, mixture of interfacial and cohesive. * *p* < 0.05, ** *p* < 0.01, *** *p* < 0.001.

The adhesion strength and work of adhesion were calculated from the maximal of the stress vs. strain curve. Each measurement was performed eight to ten times. In the box plots of the adhesion strength values (kPa) shown below (Figures 1, 3 and 4), the average stress (square and obtained value), the median stress (line), and the type of failure (I, interfacial failure; C, cohesive failure; C + I, mixture of interfacial and cohesive failure; T + C, mixture of tissue and cohesive failure) are specified for each glue as well.

### 2.4. Statistical Analysis

Statistical analysis was performed by one-way analysis of variance (ANOVA) used to determine significant difference in the result for each group of measurement followed by a post-hoc Tukey’s test (GraphPad software, La Jolla, CA, USA). A value of *p* < 0.05 was used for statistical analysis, and significance difference was set to *p* < 0.05, *p* < 0.01, and *p* < 0.001.

### 2.5. Tensile Measurements

Tensile measurements were performed with a Zwick Roell 1446 Universal Testing Machine (Zwick Roell, Ulm, Germany) equipped with a 200 N load cell. The precursor mixtures (PEG-Dop 30%, PEG-Dop 30%/Coll 0.2% or PEG-Dop 30%/Coll 0.2%/HAp 5% (*w*/*v*)) and an equivalent volume of oxidant solution (120 mM NaIO_4_ in DI water) were injected in a rectangular mold (0.8 mm × 25 mm × 75 mm) and cured for 2 h. The mold was covered by a glass slide and kept in a closed humid box to prevent from drying. For the tensile measurements, rectangular hydrogel samples were prepared, and the exact dimensions and effective length of the samples were measured before each experiment with a digital vernier scale. Tensile measurements were performed at a strain rate of 20 mm/min until failure. The Young’s modulus, the tensile strength, and strain were respectively calculated from the initial slope and the maximal stress upon failure of the stress vs. strain curve. Each measurement was performed in triplicate.

### 2.6. Scanning Electron Microscopy Imaging

The microstructures of hydrogel samples were characterized with a scanning electron microscopy (SEM) FEI Quanta 400 (FEI, Hillsboro, OR, USA). The samples PEG-Dop 15%, PEG-Dop 15%/HAp 2.5%, PEG-Dop 15%/Coll 0.1%, and PEG-Dop 15%/HAp 2.5%/Coll 0.1% (*w*/*v*) were cured between two disks of skin for adhesion measurements. After being cured for 30 min, the cured hydrogels were immersed for 10 min in DI water and then serially transitioned from water to ethanol for 20 min immersions in 20%, 40%, 60%, 80% solutions of ethanol in water, ending with overnight immersion in pure ethanol. Ethanol dehydrated hydrogels were dried in a K850 critical point dryer (LOT-QuantumDesign GmbH, Darmstadt, Germany). Prior to imaging, dried hydrogels were mounted on SEM sample stages using carbon tape and sputter-coated with a gold layer. The samples were imaged at 5 kV.

### 2.7. In Vitro Cytocompatibility Studies

Preparation of hydrogel samples: 13 mm diameter glass coverslips were cleaned with ethanol followed by 10 min oxidative plasma treatment. Cleaned substrates were immersed in a 10 mg/mL solution of dopamine buffered with Tris-HCl 10 mM to pH 8.5 for 30 min, rinsed with ultrapure water and dried under an N_2_ stream. With this treatment, a thin poly(dopamine) layer is deposited onto the glass. A quantity of 20 µL of a PEG-Dop 30%, Coll 0.2%, Hap 5% (*w*/*v*), and 120 mM NaIO_4_ mixture was deposited on a Sigmacote^®^ (Sigma-Aldrich, Taufkirchen, Germany) treated glass slide (76 mm × 26 mm) [42] and covered with the polydopamine-coated glass slide. The Sigmacote^®^ layer avoids reaction and attachment of the glue with the glass substrate. The hydrogel was cured for 1 h in a closed humid box. The formed hydrogel was then gently peeled-off from both glass surfaces, washed with PBS three times, and kept immersed in PBS until cell seeding. Live/dead and MST-1 assays were performed in triplicate.

Live/dead assay: The cytocompatibility of the substrates was performed with Live/Dead Cell Double Staining (Sigma-Aldrich) based on fluorescein diacetate (FDA) and propidium iodide (PI) by following the manufacturer’s protocol. Briefly, L929 cells (50,000 cells/well) were seeded in 24-well plates (Greiner Bio-One, Kremsmünster, Austria) overnight. The hydrogel sample was sterilized by immersion in 75% EtOH for 3 min, then introduced to the well; the cells were then incubated in the presence of the hydrogel for 24 h. After 24 h culture, the cell medium was removed and the cells were washed once with PBS. Subsequently, a working solution (30 µg/mL FDA and 40 µg/mL PI) was then added directly to the cells for 5 min, the staining solution was removed, and the cells were washed with PBS once. Subsequent imaging was performed using epifluorescence microscopy. Live cells were stained green (excitation/emission ≈490/515 nm) while dead cells appeared in red color (excitation/emission ≈535/617 nm). As control experiments, untreated cells (cells incubated in the absence of the hydrogel) were used as PI negative control (viable cells), and 0.1% Triton X-100 (TX) treated cells for 5 min were used as PI positive control (dead cells).

Cell viability assay: Cell viability tests were performed using WST-1 staining (Sigma-Aldrich) following the manufacturer´s protocol. Briefly, L929 cells (50,000 cells/well) were seeded in 24-well plates (Greiner Bio-One) overnight. The hydrogel sample was then introduced to the well and the cells were incubated in the presence of the hydrogel for 24 h. Untreated cells (cells incubated in absence of hydrogel) were used as control. After 24 h, the WST-1 reagent was diluted in cell culture medium in a 1:10 ratio and added to the cells. Metabolically active cells cleaved the stable tetrazolium salt WST-1 to a soluble formazan. After 4 h incubation, the 24-well plates were measured at wavelengths 450 and 690 nm with a plate reader (Infinite M200; Techan, Durham, NC, USA). The mean absorbance of untreated cells was defined as 100%, and the absorbance of the cells incubated with hydrogel was calculated relative to this value.

## 3. Results and Discussion

PEG-Dop with high substitution degree (>90%) and different molecular weights (5, 10, and 40 kDa) were synthesized in a 1 g scale and used for adhesion testing in different mixtures. Pull-off experiments on glued pork skin samples were established to characterize adhesion performance using a tensile testing machine [8,28]. The adhesion strength was calculated from the maximum force detected in the pull-off curves, at which the glue started detaching from the skin (interfacial failure) or breaking in two parts (cohesive failure, Supplementary Figure S3).

In preliminary experiments, the adhesion strength of PEG-Dop/NaIO_4_ mixtures at conditions reported by other groups [43] was tested for reference. PEG-Dop (15% (*w*/*v*)) with 120 mM NaIO_4_ showed an adhesive strength of 63.6 kPa. Interestingly, PEG-Dop glue predominantly failed cohesively, i.e., a crack propagated through the gluing material, while the glue–skin interface remained stable. These results suggest that the strength of the interface PEG-Dop–skin is stronger than the PEG-Dop hydrogel itself. Consequently, adhesion performance of the PEG-Dop–skin system might be improved by mechanically reinforcing the polymeric network.

The stability of PEG-Dop underwater was checked by immersing the glued skin in PBS for 30 min before performing the adhesive test. PEG-Dop showed average values of adhesion strength of 25.5 kPa, significantly lower than in dry conditions (63.6 kPa) but still remarkable when compared with commercial systems like cyanoacrylate or fibrin (Figure 1A). Mainly, the failure type was cohesive. Poly(ethylene glycol) is a highly hydrophilic polymer that can easily uptake water depending on its cross-linking degree. Water uptake softens the gel and leads to lower cohesive properties and adhesive strength.

Our results suggest that cohesively-failed PEG-Dop used in reported works [28,40,41] might offer improved adhesive performance if the mechanical properties of the cured gel were to be significantly improved without compromising the interfacial strength provided by the Dop modification. Encouraged by this finding, we explored this possibility by tuning the formulation of PEG-Dop gels following four different strategies: (i) changing the molecular weight of 4-arm PEG and polymer concentration in the gluing solution, (ii) adding HAp nanoparticles to PEG-Dop glues, (iii) mixing with Coll, and (iv) tuning the ratio between Dop and oxidant.

Adhesive tests on PEG-Dop gels with different molecular weights (Mw of 5, 10, and 40 kDa) were performed first. An increase of Mw from 5 to 10 kDa resulted in enhanced adhesion strength from 46.5 to 63.6 kPa (Figure 1B). The failure was cohesive in both cases, in spite of the net loss of the density of Dop groups available for bonding with tissue in the 10 kDa PEG. A higher Mw (40 kDa) led to a decrease in adhesion strength (47.7 kPa) and a mixture of cohesive and interfacial failure. This can be associated with the lower density of Dop groups in the 40 kDa PEG-Dop and consequent reduced number of the skin–glue covalent interactions and weakening of the interface. Therefore, for the following experiments we chose PEG-Dop (Mw of 10 kDa).

A further improvement of the adhesion strength of PEG-Dop mixtures was achieved by optimizing the final concentration of 10 kDa PEG-Dop. Tests with 5%, 10%, 15%, 20% (*w*/*v*) were performed (Figure 1C). The highest adhesion strength was found for 10% (*w*/*v*) PEG-Dop, with adhesion strength of 64.5 kPa and a cohesive failure; whereas 5% (*w*/*v*) PEG-Dop showed lower adhesion strength (43.0 kPa) and a mixture of interfacial and cohesive failure. PEG-Dop content higher than 15% did not lead to significant improvement of the adhesive properties. A PEG-Dop concentration of 15% (*w*/*v*) was selected for the following experiments, since consumption of catechol groups is expected to occur when mixing with HAp or Coll in later experiments and a minimum of 10% intact catechol groups was assumed for maximizing adhesive performance.

**Figure 2:**
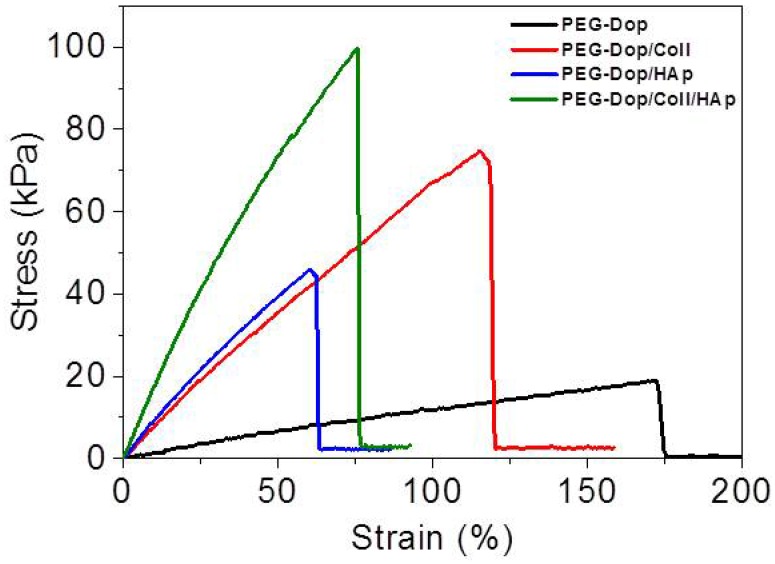
Representative tensile curves of PEG-Dop, PEG-Dop/Coll, composite hydrogel PEG-Dop/HAp, and composite mixture PEG-Dop/Coll/HAp. Concentration of the different components is as follows: 15% (*w*/*v*) PEG-Dop, 2.5% (*w*/*v*) HAp, 0.1% (*w*/*v*) Coll.

**Figure 3:**
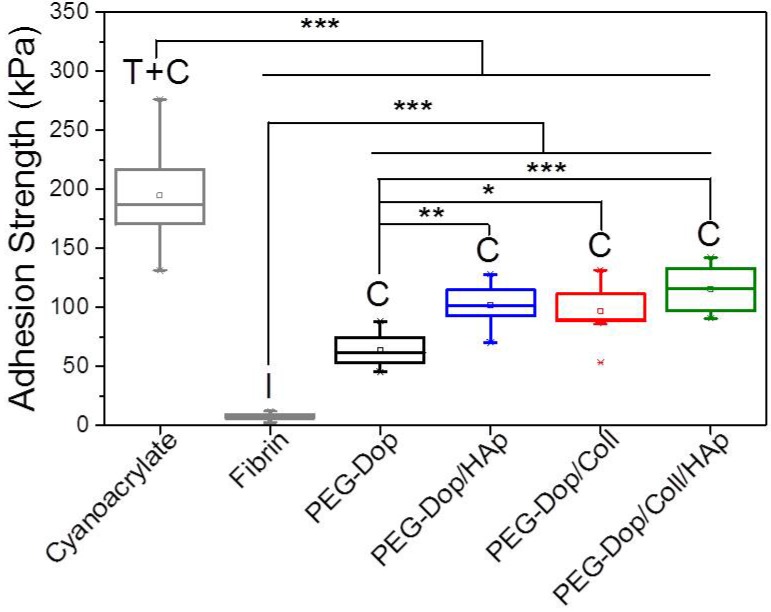
Adhesion strength values of PEG-Dop adhesives (15% (*w*/*v*) PEG-Dop, 2.5% (*w*/*v*) HAp, 0.1% (*w*/*v*) Coll), compared with commercial cyanoacrylate and fibrin glues on dry skin. Failure type: I, interfacial; C, cohesive; T + C, mixture of tissue and cohesive. * *p* < 0.05, ** *p* < 0.01, *** *p* < 0.001.

Addition of nanoparticles able to strongly interact with the polymer chains of the hydrogel is an effective way to reinforce mechanical properties and can lead to strong nanocomposite hydrogels [21,44,45,46,47,48,49]. When the interaction between the nanoparticle surface and the polymer chain is strong, the nanoparticles act as multivalent crosslinkers within the polymer network and enhance its mechanical strength. We applied this strategy to PEG-Dop hydrogels by mixing biocompatible HAp nanoparticles [50]. According to literature reports, the Dop end-groups in the PEG chain are expected to coordinate with the ionic surface of the HAp nanoparticles and reinforce the network [51,52,53]. In fact, tensile tests of PEG-Dop (15%)/HAp (2.5%) mixtures showed a >6-fold increase in the Young’s modulus in comparison with the PEG-Dop hydrogel without added nanoparticles (Figure 2 and Table 1). Adhesion testing of PEG-Dop (15% (*w*/*v*)) formulations with increasing ratios of HAp particles (0, 0.5, 1, 2.5, and 5% (*w*/*v*)) gave increasing adhesion strengths (Figure 1D and Figure 3). The type of adhesive failure remained cohesive for all samples. In conclusion, the addition of HAp nanoparticles led to a higher mechanical resistance of the hydrogel and to an increase in the final adhesive performance at concentrations up to 2.5%. It is important to note that the positive effects of the addition of HAp in terms of mechanical reinforcement are presumably counteracted by lower cross-linking degrees of the PEG matrix, since Dop units become coordinated to the HAp surface and are no longer available for self-reaction. This might be the reason why HAp concentrations higher than 2.5% did not further improve the adhesive performance.

We then tested the ability of PEG-Dop to form homogeneous mixtures with collagen type I at increasing concentrations (0.05–0.2% (*w*/*v*)). Collagen type I forms physical networks at pH 7–7.4 and 37 °C [27]. In the mixture PEG/Coll, the catechol units might also react with the nucleophiles (like amine groups) of the collagen and both networks are expected to be covalently interconnected. Homogeneous networks were obtained under conditions specified in the Materials and Methods section. The polymer mixture significantly enhanced the tensile strength of the PEG-Dop hydrogel, as reflected in the tensile test in Figure 2. The adhesive performance was also significantly improved. The adhesion strength of the PEG-Dop/Coll increased from 63.6 kPa (0% Coll) to 89.3 and 97.1 kPa when adding 0.05% and 0.1% Coll content (Figure 1E and Figure 3). Pure collagen (0.2% (*w*/*v*)) gave a very low adhesion strength of 2.9 ± 1.4 kPa and failed interfacially (data not shown). The PEG-Dop/Coll mixtures including Coll content higher than 0.2% showed lower adhesion strengths (69.6 kPa), which we associate with the difficult solubilization of Coll at ≥0.4% concentrations, and consequent poor mixing and inhomogeneity of the PEG-Dop/Coll network. All adhesives showed cohesive failure, indicating that the interfacial interaction of Dop units with the skin was retained in the mixed hydrogel with collagen, in spite of catechol groups being eventually consumed by reaction with collagen and not by reaction with the tissue.

In an effort to push cohesiveness further, 2.5% (*w*/*v*) HAp nanoparticles were added to the PEG-Dop 15%/Coll 0.1% hydrogel. The Young’s modulus obtained for PEG-Dop, PEG-Dop/Coll and PEG-Dop/Coll/HAp was 15.2, 86.9, and 181.5 kPa, respectively (Table 1), confirming the significant mechanical reinforcement by the mixing of HAp nanoparticles in the hydrogel. The PEG-Dop/Coll/HAp hydrogel showed a higher tensile strain (ultimate stress upon failure) than the PEG-Dop/HAp mixture due to the high elasticity of Coll (Table 1). In other words, collagen does not only enhance the strength of hydrogel, but also improves the strain loss induced by addition of HAp nanoparticles. The composite showed the highest adhesion strength (115.5 kPa). Note that this value is 15.4-fold higher than in fibrin, and represents ca. 60% of the adhesion strength of the cyanoacrylate glue (Figure 3). In summary, the incorporation of Coll and HAp nanoparticles in the PEG-Dop hydrogel significantly improved the cohesion and skin adhesion properties of the system. Importantly, the cohesive properties can be varied by tuning the Coll and HAp concentrations, to possibly match the properties of the different tissues for application. In all cases, cohesive failure was observed, indicating that there is still room for further improvement of the adhesive performance with other hydrogel compositions for load-bearing application, i.e., by using double networks incorporating dissipative elements [54,55,56]. This possibility will be studied in the future.

According to a previous report [43], the mechanical properties and curing speed of PEG-Dop are highly affected by both the alkalinity of the oxidant solution and the ratio of Dop vs. oxidant applied; this can impact the adhesive performance. In a final attempt to improve the performance of the hydrogels, we tested the adhesion strength of PEG-Dop glues at a Dop:oxidant ratio of 1:1; when the oxidant was added either in DI water or in 0.4 M NaOH solution. Adhesion strength in DI water was significantly higher (63.6 kPa, final pH of the mixture was 6) than in basic conditions (4.3 kPa, final pH of the mixture was 12). Failure type also changed from cohesive (in water) to interfacial (in NaOH) (Supplementary Figure S4). We attribute this difference to the fast curing speed observed when using the oxidant at a higher pH. The cross-linking of the system occurred immediately upon mixing, before the gel was placed on the skin and, therefore, a very small number of catechol groups were available for gluing to skin and reinforcing the interface.

The effect of the Dop:oxidant ratio was tested with the PEG-Dop/HAp/Coll system. Oxidant concentrations of 30, 60, 120, 180, 240 mM were used, which correspond to a Dop:oxidant ratio of 4:1, 2:1, 1:1, 1:1.5, and 1:2, respectively. At 30 mM oxidant concentration, no stable hydrogel was formed. At increasing oxidant concentrations of 60–240 mM, an increase in the adhesive strength up to 126.0 kPa and a change in the failure type from interfacial to cohesive were observed (Figure 1F). The oxidant concentration affects the ratio of catechol and quinone groups in the curing gel available for reaction with the different components, PEG, Coll, or HAp. The polymerization speed also increased with the oxidant concentration. Gelation time is a relevant parameter to medical applications, and has to be adjusted to the specific surgery conditions [40,43]. For the following experiments we used 120 mM oxidant.

**Figure 4:**
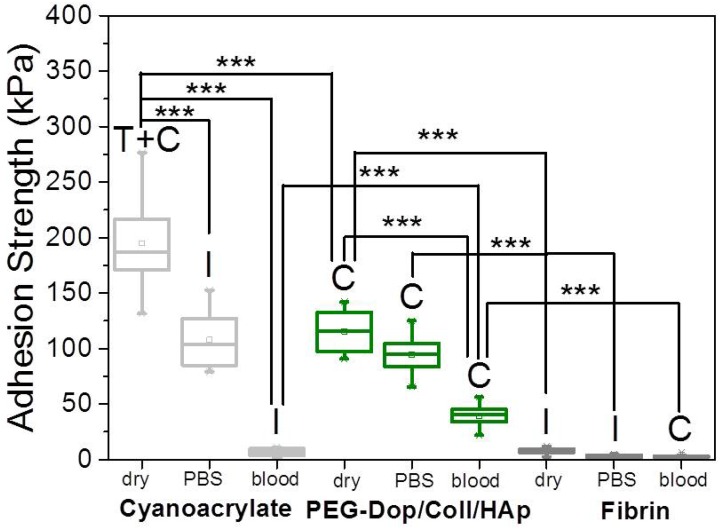
Adhesion strength values of PEG-Dop adhesives on “dry” skin, and “wet” skin (either with phosphate-buffered saline (PBS) or with blood) compared with commercial tissue glues. Failure type: I, interfacial; C, cohesive; T + C, mixture of tissue and cohesive. *** *p* < 0.001.

In a real application, tissue adhesives need to perform in the presence of body fluids. Therefore, the adhesive strength of prospective tissue glues should be tested in the presence of PBS or blood. We characterized the adhesion performance of PEG-Dop/Coll/HAp on wet skin (after immersion in PBS for 2 min) and skin covered with blood (containing heparin anticoagulants) and compared with the performance on dry skin (Figure 4). PEG-Dop/Coll/HAp lost only 18% of its adhesion strength on wet skin (95 kPa). Importantly, this value was close to the adhesion strength of commercial cyanoacrylate glue to wet skin (108.1 kPa). It is worth mentioning that failure remained cohesive on our composite hydrogel on wet skin, while failure in cyanoacrylate changed to be interfacial. We attribute the loss of adhesive performance of cyanoacrylate in the presence of water to its hydrophobic nature, which prevents tissue contact and possibly penetration when water is in between. Notably, PEG-Dop/Coll/HAp significantly outperformed cyanoacrylate in adhesion tests in the presence of blood. Our composite hydrogel showed adhesion strength of 38.9 kPa, which is ≈5.6-fold higher than adhesion strength of cyanoacrylate (6.9 kPa) under comparable conditions. Failure remained cohesive in the composite gel, indicating that the strong interfacial interaction between the dopamine-modified system and the bloody tissue was retained, and possibly further reinforced by attractive interactions between the collagen and the blood proteins. This was also found in fibrin glue, which changed from interfacial to cohesive failure when measured on skin in the presence of PBS or blood. Fibrin–blood interactions during fibrinogenesis and clot formation seem to positively influence adhesion of fibrin glues to skin. However, the adhesion strength of fibrin was ≈13.4-fold lower than the adhesion strength of PEG-Dop/Coll/HAp under the same measuring conditions. In summary, PEG-Dop-based tissue glues can lead to customized systems with application-specific cohesive and adhesive properties, including mechanically demanding uses.

**Figure 5:**
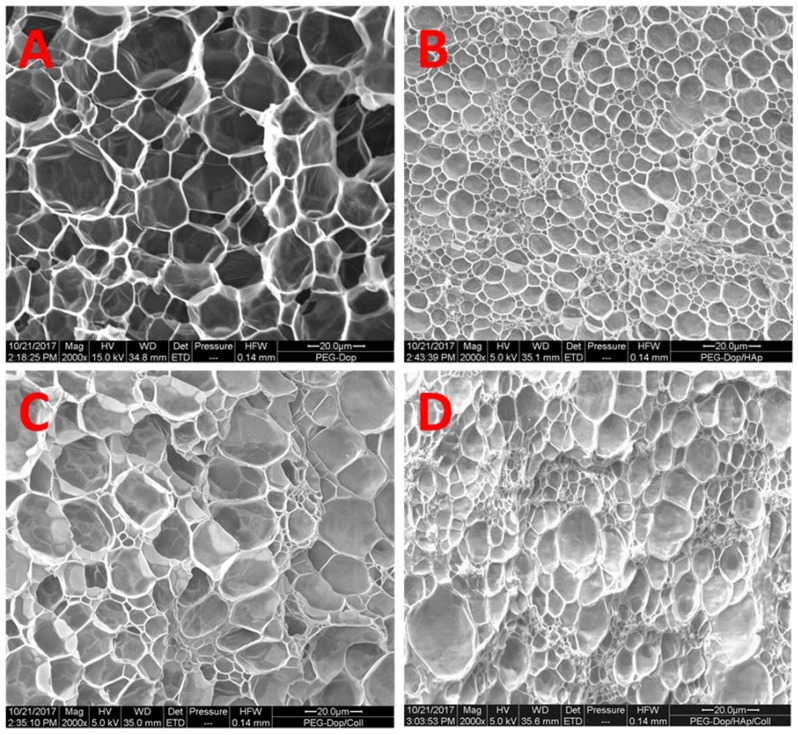
Scanning electron microscopy (SEM) images of the bulk structure of (**A**) PEG-Dop; (**B**) PEG-Dop/HAp; (**C**) PEG-Dop/Coll; (**D**) PEG-Dop/HAp/Coll. Concentration of the different components was: 15% (*w*/*v*) PEG-Dop, 2.5% (*w*/*v*) HAp, 0.1% (*w*/*v*) Coll. In all cases, scale bar: 20 μm.

It should be noted that the reinforcement mechanism of collagen type I in our mixtures is not clear. An important feature of collagen type I is its possibility to form fibrils, and fibrillar protein structures can mechanically reinforce gels. In order to test if fibril formation occurred in our gels, the morphology of bulk PEG-Dop, PEG-Dop/HAp, PEG-Dop/Coll, and PEG-Dop/Coll/HAp hydrogels was characterized by SEM. Samples cured for 30 min were subjected to critical point drying and imaged by SEM. Porous structures were observed in all hydrogels (Figure 5). Changes in pore sizes were observed with added HAp, Coll, or both to the polymer mixture. Addition of HAp resulted in a more compact structure (smaller pores). In PEG-Dop/HAp hydrogel (Figure 5B), HAp appeared to be not homogeneously distributed, with the pores around HAp smaller than pores in other places. PEG-Dop/Coll hydrogels (Figure 5C) showed similar pore sizes to PEG-Dop, although size distribution was broader. Individual collagen fibers were not observed in the mixture, which typically have a striated morphology [57]. The final formulation showed an intermediate pore geometry (Figure 5D) which, according to the mechanical tests, was beneficial to the improvement of strength and maximum strain of the hydrogel. Future studies will change the gel preparation method and explore possible beneficial properties of fibril formation for mechanical reinforcement of the gels.

Finally, we tested the cytocompatibility of the PEG-Dop/Coll/HAp hydrogel. Fibroblasts were cultured for 24 h either in the presence or absence of a thin hydrogel, and live/dead and WST-1 assays were performed to determine cell viability (Supplementary Figure S5). Cells incubated in presence of hydrogel remained viable (96%, in relation to a control of cells incubated in absence of hydrogel). These results are in agreement with those reported on the literature for PEG-Dop, which has proved biocompatible in vitro and in vivo [8,37].

## 4. Conclusions

Research in tissue glues has mainly been focused on improving the reactivity between artificial polymers and tissue with effective and biocompatible chemistries using in situ forming hydrogels. However, adhesive failure in hydrogels is often of a cohesive nature as a consequence of their swelling and low mechanical stability. Consequently, efforts in the design of more mechanically robust hydrogel backbones can significantly increase adhesive performance of hydrogel-based tissue glues. For this purpose, reinforced hydrogels offer interesting possibilities. In our work, mechanical reinforcement of PEG-Dop with collagen and by adding HAp nanoparticles lead to a significant increase in adhesive performance. It is important to note that these systems surpass by far the adhesion strength of commercially available products and reported adhesive systems in the presence of blood. The biocompatibility and the optional tunable degradability of PEG-Dop gels including peptide sequences in the backbone make this material’s design promising for the real application of bioinspired tissue glues in medicine. Finally, the cohesive properties of the hydrogel might be further reinforced by introducing dissipative components in the backbone, as recently demonstrated for tough hydrogels, by integrating double network architectures, or by allowing fibrillar collagen to form an internal physical network. These possibilities will be the subject of further studies by our group.

## Figures and Tables

**Table 1:** Tensile tests of PEG-Dop, PEG-Dop/Coll, composite hydrogel PEG-Dop/HAp and composite mixture PEG-Dop/Coll/HAp.

	Young’s Modulus (kPa)	Tensile Strength (kPa)	Strain (%)
PEG-Dop	15.2 ± 4.7	18 ± 1.3	182.3 ± 42.7
PEG-Dop/Coll	86.9 ±18.8	66.7 ± 6.5	100.9 ± 21.4
PEG-Dop/HAp	96.6 ± 4.9	45.2 ± 1.2	64.7 ± 3.0
PEG-Dop/Coll/HAp	181.5 ± 39.3	88.5 ± 9.5	61.3 ± 20.6

Concentration of the different components is as follows: 15% (*w*/*v*) PEG-Dop, 2.5% (*w*/*v*) HAp, 0.1% (*w*/*v*) Coll. Mean value ± standard deviations are given, *n* = 3.

## Supplementary Materials

The following are available online at www.mdpi.com/2313-7673/2/4/23/s1. Synthesis of PEG-Dop, Figure S1: Proton nuclear magnetic resonance (^1^H-NMR) spectra of PEG-Dop polymers, Figure S2: Pictures of the set-up used for the adhesion strength measurements, Figure S3: Picture of pork skin samples bonded with PEG-Dop adhesive hydrogel and fractured during the adhesion test, Figure S4: Adhesion strength values of 15% (*w*/*v*) PEG-Dop mixtures with 120 mM oxidant in 0.4 M NaOH solution vs. in DI water, Figure S5: Cytotoxicity experiments.
