# Supplementary material for: Mechanically Reinforced Catechol-Containing Hydrogels with Improved Tissue Gluing Performance
Source: Biomimetics (Basel). 2017 Nov 13;2(4):23. doi: 10.3390/biomimetics2040023 (PMC6352675; doi:10.3390/biomimetics2040023)
Supplement: Supplementary file 1 [file biomimetics-02-00023-s001.pdf]

*Biomimetics* **2017**, *2*, 23

# Supplementary Materials: Mechanically Reinforced Catechol-Containing Hydrogels with Improved Tissue Gluing Performance

**Jun Feng [1,3†], Xuan-Anh Ton [2,†], Shifang Zhao [1,3], Julieta I. Paez [1] and Aránzazu del Campo [1,3,*]**

[1] INM – Leibniz Institute for New Materials, Campus D2 2, 66123 Saarbrücken, Germany; Jun.Feng@leibniz-inm.de (J.F.); shifang.zhao@leibniz-inm.de (S.Z.); julieta.paez@leibniz-inm.de (J.I.P.)

[2] Max-Planck-Institut für Polymerforschung, Ackermannweg 10, 55128 Mainz, Germany; xuananh.ton@gmail.com

[3] Chemistry Department, Saarland University, 66123 Saarbrücken, Germany

* Correspondence: aranzazu.delcampo@leibniz-inm.de; Tel.: +49-(0)681-9300-510; Fax: +49-(0)681-9300-223

† These authors contributed equally to this work.

**Supplemental Materials and Methods**

**Synthesis of PEG-Dop:** A protocol previously reported by our group was followed [1,2]. Dopamine (45.6 mg, 0.24 mmol) was reacted for 15 min with *N*-methylmorpholine (44 μL, 0.40 mmol) in 2.0 mL of anhydrous dimethylformamide (DMF) under agitation in argon atmosphere. 4-arm PEG succinimidyl carboxymethyl ester (PEG-NHS, Mw of 5, 10, or 40 kDa) (400 mg, 0.040 mmol) in 3.0 mL of anhydrous DMF was added and the reaction mixture was stirred overnight at room temperature under argon. The crude product was concentrated under reduced pressure, dissolved in deionized water (pH 4.5), purified by dialysis against deionized water (pH 4.5) using membrane tubing (Mw cut-off of 3.5 kDa) and lyophilized. The degree of catechol substitution was obtained by $^{1}$H-NMR (Bruker Advance III, 300MHz, Karlsruhe, Baden-Württemberg, Germany) end-group determination in CD2Cl2 (ca. 13 mg/mL, Supplementary Figure S1). The integral of the signal corresponding to the PEG backbone (3.70–3.50 ppm) was set to 110, 220, or 880 H (corresponding to Mw of 5, 10, and 40 kDa, respectively) and compared with the integral of the catechol protons (3H of aromatic ring, 6.80– 6.50 ppm). About 80–95% NHS groups were substituted by catechol groups in all polymer batches.

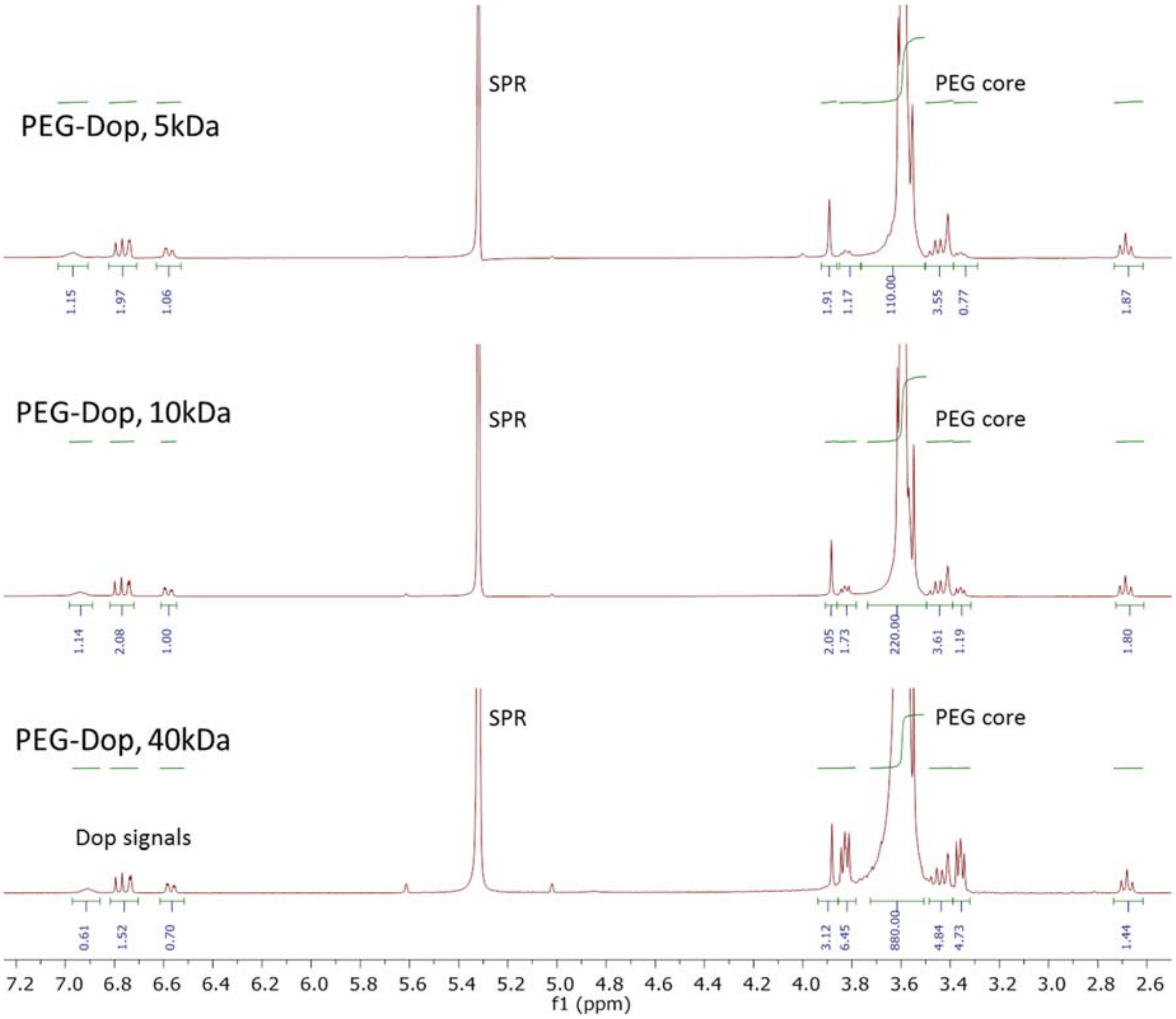


**Figure S1.** Proton nuclear magnetic resonance ($^1$H-NMR) spectra of PEG-Dop polymers. SPR: Solvent residual peak (DCM).

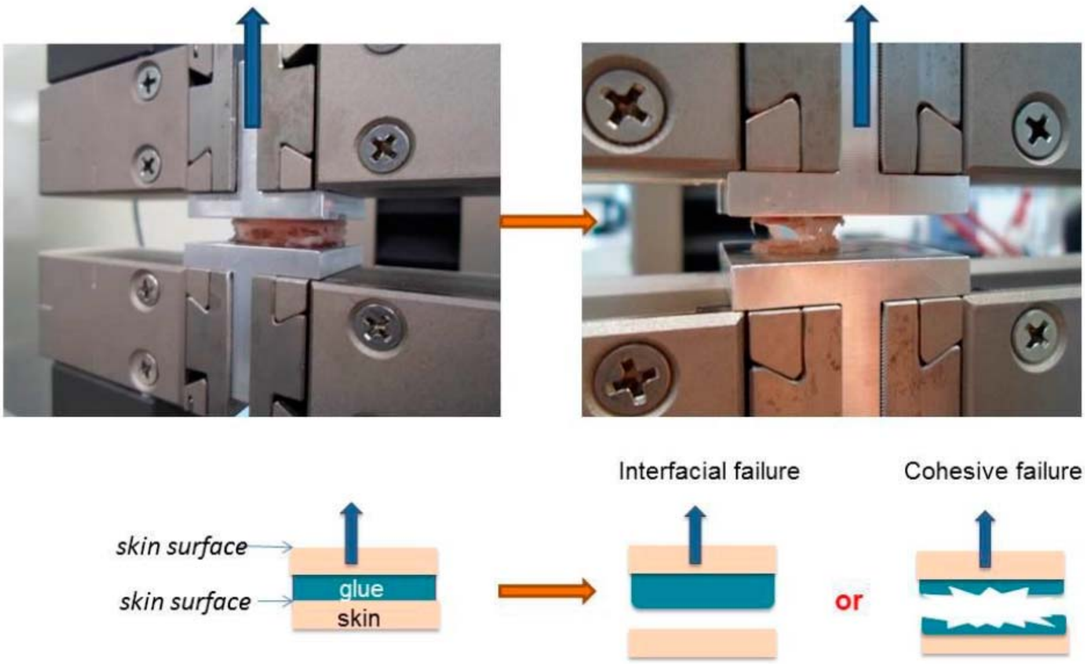


**Figure S2.** Pictures of the set-up used for the adhesion strength measurement. The sample was elongated at a strain rate of 1 mm/min until it failed adhesively (the polymer detaches from the skin) or cohesively (the polymer breaks in two).

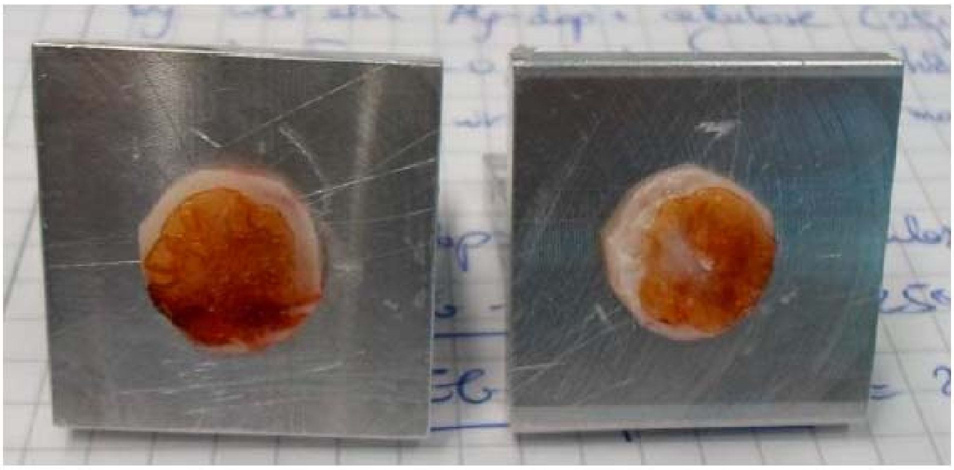

**Figure S3.** Picture of pork skin samples bonded with PEG-Dop adhesive hydrogel and fractured during the adhesion test. Cohesive failure is revealed by the presence of PEG-Dop hydrogel (brown) on the two pieces of skin.

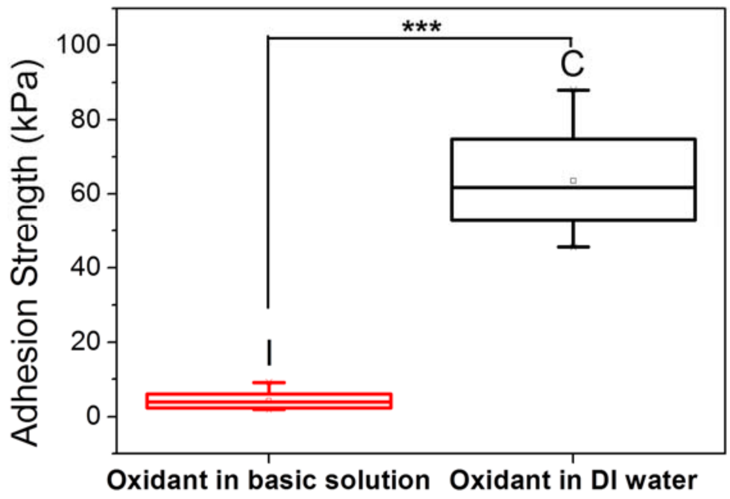


**Figure S4.** Adhesion strength values of 15% (*w*/*v*) PEG-Dop mixtures with 120 mM oxidant in 0.4 M NaOH solution vs. in DI water. Type of failure: I, interfacial; C, cohesive.

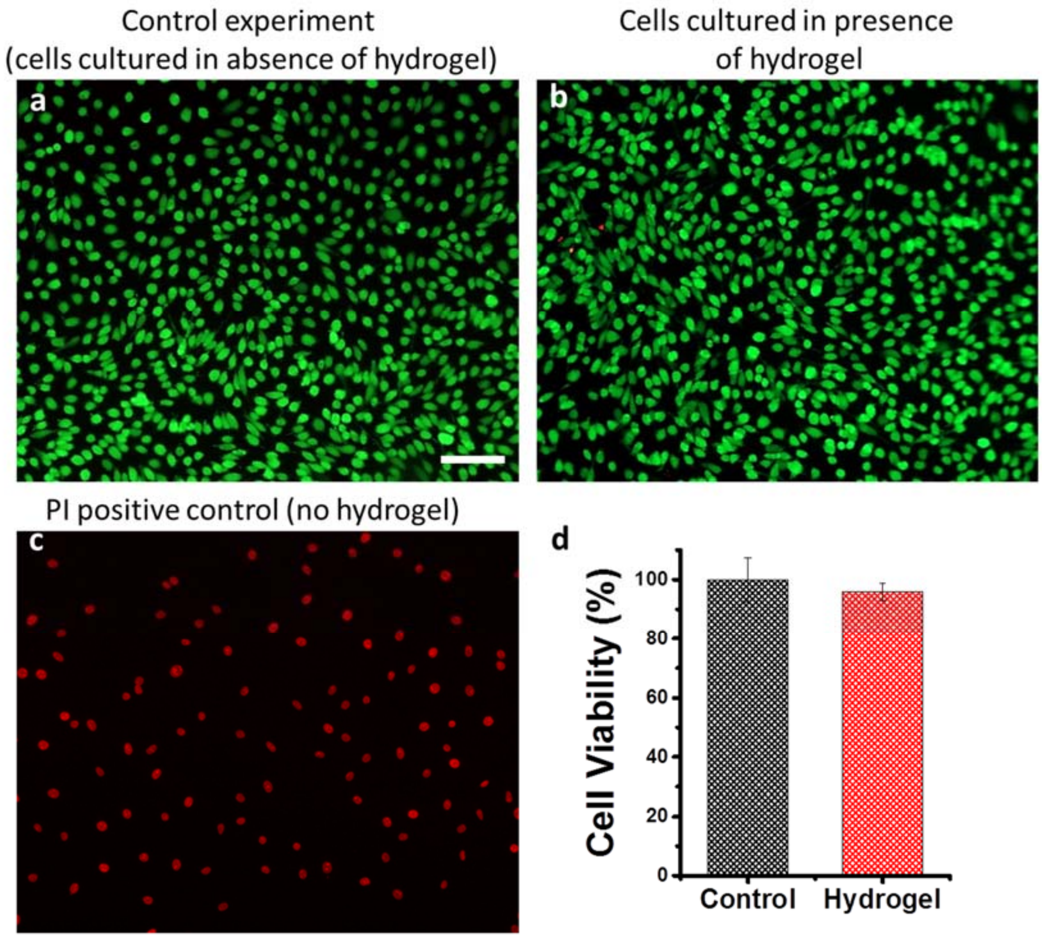


**Figure S5.** Cytotoxicity experiments. (**a**–**c**) Typical live/dead assay images of cells treated with different condition for 24 h. (**a**) Control experiment (cells cultured in the absence of hydrogel) were used as PI negative control, (**b**) cells incubated in the presence of hydrogels, and (**c**) cells treated with 0.1% Triton X-100 (2% TX) as PI positive control. Live cells are labeled in green and dead cells in red. Scale bar: 100 μm. (**d**) Cell viability of cells cultured in the absence of hydrogel (control, 100%) and in the presence of hydrogel (calculated in relation to the control), by WST-1 assay.

## References


1. García-Fernández, L.; Cui, J.; Serrano, C.; Shafiq, Z.; Gropeanu, R.A.; Miguel, V.S.; Ramos, J.I.; Wang, M.; Auernhammer, G.K.; Ritz, S.; et al. Antibacterial strategies from the sea: Polymer-bound Cl-catechols for prevention of biofilm formation. *Adv. Mater.* **2013**, *25*, 529–533.
2. Paez, J.I.; Ustahüseyin, O.; Serrano, C.; Ton, X.-A.; Shafiq, Z.; Auernhammer, G.K.; d'Ischia, M.; del Campo, A. Gauging and tuning cross-linking kinetics of catechol-PEG adhesives via catecholamine functionalization. *Biomacromolecules* **2015**, *16*, 3811–3818.
